# The interval between oocyte retrieval and frozen-thawed blastocyst transfer does not affect the live birth rate and obstetrical outcomes

**DOI:** 10.1371/journal.pone.0206067

**Published:** 2018-10-19

**Authors:** Mathilde Bourdon, Pietro Santulli, Chloé Maignien, Khaled Pocate-Cheriet, Asim Alwohaibi, Louis Marcellin, Sarah Blais, Charles Chapron

**Affiliations:** 1 Université Paris Descartes, Sorbonne Paris Cité, Faculté de Médecine, Assistance Publique–Hôpitaux de Paris (AP–HP), Hôpital Universitaire Paris Centre, Centre Hospitalier Universitaire (CHU) Cochin, Department of Gynaecology Obstetrics II and Reproductive Medicine, Paris, France; 2 Institut Cochin, Laboratoire d’immunologie, Université Paris Descartes, Sorbonne Paris Cité, Paris, France; 3 Université Paris Descartes, Sorbonne Paris Cité, Faculté de Médecine, Assistance Publique–Hôpitaux de Paris (AP- HP), Hôpital Universitaire Paris Centre, Centre Hospitalier Universitaire (CHU) Cochin, Service d'Histologie-Embryologie-Biologie de la Reproduction, Paris, France; 4 Institut Cochin, Département de “Génetique, Développement et Cancer”, Université Paris Descartes, Sorbonne Paris Cité, Paris, France; University Hospital of Münster, GERMANY

## Abstract

**Background:**

The ‘Freeze all’ strategy, which consists of cryopreservation of all embryos after the ovarian stimulation has undergone extensive development in the past decade. The time required for the endometrium to revert to a prestimulation state after ovarian stimulation and thus the optimal time to perform a deferred embryo transfer after the stimulation has not been determined yet.

**Objective:**

To investigate the impact of the time from oocyte retrieval to frozen-thawed blastocyst transfer (FBT) on live birth rate (LBR), obstetrical and neonatal outcomes, in ‘Freeze-all’ cycle.

**Materials and methods:**

We conducted a large observational cohort study in a tertiary care university hospital including four hundred and seventy-four first autologous FBT performed after ovarian stimulation in ‘freeze all’ cycles. Reproductive outcomes were compared between FBT performed within the first menstrual cycle after the oocyte retrieval (‘cycle 1’ group) or delayed FBT (‘cycle ≥ 2’ group). The main Outcome Measure was the Live birth rate.

**Result(s):**

A total of 188 FBT were included in the analysis in the ‘cycle 1’ group and 286 in the ‘cycle ≥ 2’ group. No significant differences were found between FBT performed within the first menstrual cycle after oocyte retrieval (the ‘cycle 1’ group) and delayed FBT (the ‘cycle ≥ 2’ group) in terms of the live birth rate [59/188 (31.38%) vs. 85/286 (29.72%); p = 0.696] and the miscarriage rate [20/82 (24.39%) vs. 37/125 (29.60%), respectively; p = 0.413]. The obstetrical and neonatal outcomes were also not significantly different between the two groups.

**Conclusion:**

Our study did not detect statistically significant differences in the LBR for FBT performed within the first menstrual cycle after oocyte retrieval versus FBT following subsequent cycles. Embryo-endometrium interaction after a FBT does not appear to be impaired by potential adverse effects of COS whatever the number of cycle between oocyte retrieval and embryo transfer.

## Introduction

Controlled ovarian stimulation (COS) is an essential step in achieving a multi-follicular development with ART procedures such as in vitro fertilization / intra cytoplasmic sperm injection (IVF/ICSI). A growing concern is that COS, which generates a ‘supraphysiological’ level of estradiol, may alter endometrial receptivity [1–5]. COS could lead to an advance in the maturation of the endometrium and therefore a lack of embryo-endometrium synchrony. This could disturb implantation, early development of the embryo, and consequently induce a reduction in assisted reproductive technology (ART) success rates [5,6]. In addition, there is increasing evidence of a negative impact of COS on obstetrical outcomes [7–9]. After a fresh embryo transfer performed immediately after the COS, the risks of premature deliveries and low birth weights appear to be higher compared to pregnancies resulting from frozen embryo transfers performed well after the COS [10–12].

In order to overcome this problem, the ‘freeze all’ strategy, which consists of cryopreservation of all embryos after the COS, allows the embryo transfer to be undertaken in cycles subsequent to the COS [5,13,14]. This deferred embryo transfer (Def-ET) strategy has undergone extensive development in the past decade. A major difference between fresh and deferred embryo transfers is the absence of COS immediately prior to the embryo transfer. Although there are still discrepancies in this regard in the literature, there are indications that this strategy increases pregnancy rates and that it improves obstetric and neonatal outcomes relative to fresh embryo transfers by limiting the negative impact of COS on implantation [11,15,16].

Although there are numerous reports regarding adverse effects of COS on ART outcomes, the time required for the endometrium to revert to a prestimulation state after COS and thus the optimal time after ovarian stimulation to perform a deferred embryo transfer has not been determined yet. A limited number of studies indicate that endometrial receptivity is restored after the first withdrawal bleeding following retrieval of oocytes [17–19]. The authors concluded that performing a frozen embryo transfer during this cycle, immediately after the COS, as compared to a transfer during subsequent cycles is not associated with a negative impact on the pregnancy rate. However, these preliminary results need to be confirmed, particularly in terms of obstetrical and neonatal outcomes.

In order to investigate what is the optimal interval between ovarian stimulation and frozen embryo transfer, and in order to discern a potential influence on reproductive outcomes in the ‘freeze all’ strategy, we undertook a cohort study to compare pregnancy and obstetrical outcomes in patients who underwent a FBT within the first menstrual cycle after the oocyte retrieval versus patients who had a FBT after subsequent cycles.

## Materials and methods

### Study population and inclusion criteria

We conducted an observational cohort study between November 2012 and December 2015 in a single ART unit at a university-based reproductive medicine center, including the first autologous frozen-thawed blastocyst transfer performed after oocyte retrieval in the setting of a Def-ET strategy.

Inclusion in this cohort study required that the following criteria were met: (a) ART with IVF or ICSI, (b) age ≤ 42 years at the time of the oocyte retrieval, and (c) an autologous frozen-thawed blastocyst transfer as part of a Def-ET strategy.

The exclusion criteria were: (a) cancelled embryo transfers, (b) FBT derived from vitrified oocyte procedures, and (c) cycles with missing data.

Only the first embryos transferred immediately after the oocyte retrieval were retained for the study.

Two groups were generated based on the number of cycles after the COS: (a) a group for which the FBT took place within the first menstrual cycle after the oocyte retrieval (‘cycle 1’) and (b) a group for which the FBT took place following one or more menstrual cycles (‘cycle ≥ 2’).

This study was approved by the National Data Protection Authority (Commission Nationale de l’Informatique et des Libertés, CNIL n° 1988293 v 0) on the 5^th^ of September 2016.

### Ovarian stimulation

The following controlled ovarian stimulation protocols were used according to our institutional clinical protocols, with 150–450 IU/day of recombinant FSH (Puregon, MSD, Courbevoie, France; Gonal-F, Merck-Serono, Lyon, France) and urinary FSH (hMG, Menopur, Ferring Pharmaceuticals, Gentilly, France): (a) a GnRH antagonist protocol, (b) a long agonist protocol, and (c) a short agonist protocol [20]. Gonadotropin doses and the type of COS protocol were determined according to the individual patient’s characteristics. Final oocyte maturation was triggered when ≥ 3 ovarian follicles of ≥ 17 mm were visible by ultrasound and when E2 levels were ≥ 1,000 pg/mL. Final oocyte maturation was achieved using either a single injection of 0.2 mg of GnRH agonist (Triptoreline, Decapeptyl, Ibsen France), or by 250 μg of recombinant hCG (rhCG, Ovitrelle, Serono, France), according to the COS protocol. Oocyte retrieval was performed 35–36 h later by transvaginal aspiration under ultrasound guidance. Indications for deferred frozen-thawed embryo transfers were as detailed previously (Bourdon *et al*., 2016).

### Embryo culture, cryopreservation and thawing

For prolonged cultures, embryos were transferred into a 50 μL droplet of one-step Global culture medium (LifeGlobal, USA) and cultured until day-5 or day-6 at 37°C in an atmosphere of 5% CO_2_, 5% O_2_, and 90% N_2_. The culture medium was changed on day-3. The embryo morphology was evaluated on the morning of day-5 and day-6. Blastocysts were scored according to the grading system of Gardner and Schoolcraft [21] and considered eligible for cryopreservation on day-5 or day-6 if they qualified as full (B3) or expanded (B4-5) blastocysts with a type A-C inner cell mass and/or a type A-C trophectoderm. Blastocysts that did not meet these criteria on day-5 were maintained in culture and re-examined on day-6. Blastocysts with a type “C” inner cell mass (ICM) and a type “C” trophectoderm were not cryopreserved, regardless of their degree of expansion and the day of observation (day-5 or day-6). A good-quality embryo was defined as a B3-B4 or a B5 embryo ≥ BB (AA, AB, BA, BB) according to the grading scale proposed by Gardner [21].

Embryo vitrification was performed using closed Cryo Bio System vitrification (CBS-VIT) High Security (HS) straws in combination with DMSO-EG-S as the cryoprotectant (Irvine Scientific Freeze Kit) at the blastocyst stage after 5 or 6 days in culture. All of the embryos were thawed using an Irvine Scientific Thaw Kit. Blastocysts were warmed on the day of the transfer. When the warmed blastocyst had < 50% intact cells, an additional blastocyst was warmed, if available. If the blastocyst was > 50% intact, expansion and re-expansion were assessed 2–3 hours later.

### Endometrial preparation prior to embryo transfer

Women received an E2 regimen that was delivered transdermally (0.2 mg/day, simultaneously through two Vivelledot 100 μg systems, Novartis Pharma SA, Rueil-Malmaison, France) or orally (4 mg twice daily, Provames, Sanofi Aventis, Paris, France) for a minimum of two weeks, after which the patients were examined in order to schedule the embryo transfer. The first day of the estradiol administration was considered to be the first day of the menstrual cycle. Endometrial thickness and progesterone levels were assessed the day before the progesterone administration. If conditions were appropriate (e.g. endometrium thickness ≥ 6 mm and progesterone < 1.5 ng/mL), embryo transfer was performed on the 5^th^ day of progesterone exposure. Vaginal progesterone treatment was initiated at a dose of 200 mg three times daily (Utrogestan, Besins International, Montrouge, France). Women who became pregnant by these procedures continued to be treated with progesterone and E2 at the same dose until 12 weeks of gestation.

### Data analysis and statistics

The general characteristics of the patients in both of the groups were recorded prospectively during face-to-face interviews prior to the embryo transfer. The following data were collected: age at retrieval (years), smoking habits, body mass index (BMI, calculated as weight (kg)/[height (m)]^2^), type of infertility (primary, secondary), number of previous IVF/ICSI cycles, day-3 FSH, antral follicle count (AFC) and anti-Müllerian hormone (AMH) levels, as well as causes for infertility (e.g. an ovulation disorder, male factor, tubal factor, endometriosis, idiopathic, diminished ovarian reserve, or more than one etiology).

The primary outcome was the live birth rate, which was defined as delivery of a viable infant at 24 weeks or more of gestation [22]. Secondary outcomes were clinical pregnancy, ongoing pregnancy, miscarriage, delivery, and neonatal outcomes.

Clinical pregnancy rates (cPR) were determined by ultrasonographic documentation of at least one fetus with a heart beat at 6–7 weeks of gestation [22], the ongoing pregnancy rate (oPR) was defined as the sonographic detection of one or more intrauterine fetuses with a positive heartbeat at 12 weeks of gestation [23], and early pregnancy loss was defined as a spontaneous pregnancy demise at less than 10 weeks of gestational age [24].

The delivery and perinatal outcomes were: gestational age at delivery, defined as preterm birth (< 37 weeks of gestation) and post-term birth (> 41 weeks of gestation); low birth weight (< 2,500 g); high birth weight (≥ 4,000 g); and cesarean or vaginal delivery, [25,26].

Z-scores allow a measurement to be compared with the expected measurement based on a set of reference values [27]. Z-scores were determined using the formula: Z-score = (X_GA_ − M_GA_)/SD_GA_, where X_GA_ is the value measured at a given gestational age, M_GA_ is the expected value at this gestational age according to the reference chart, and SD_GA_ is the SD of the expected value [27]. Z-scores were determined after adjusting for the gestational age and the child’s gender.

All of the data were compiled in a digital database and analyzed using IBM SPSS Statistics version 23.0 software (SPSS Inc. Headquarters, 233 S. Wacker Drive, 11^th^ floor, Chicago, Illinois 60606, USA). A *p-*value < 0.05 was considered to be statistically significant. For univariate statistical analysis, we used the following tests: Pearson’s χ2 test or Fisher’s exact test for qualitative variables and a Student’s t-test or Mann-Whitney test for quantitative variables, as appropriate. To identify potential confounding variables that could be independently associated with live birth rate, we performed a logistic regression analysis. Potential confounding factors found to be statistically significant at the threshold of *p* ≤ 0.20 in univariate analysis were tested in a multiple logistic regression model. Maternal age (> 35 years old), the number of previous IVF cycles, the peak estradiol levels (pg/mL) at triggering, the type of triggering, the embryo quality, and the FBT cycle (‘cycle ≥ 2’ vs. ‘cycle 1’) were included in the analysis.

Backward stepwise selection was used to retain variables with a *p-*value of < 0.05 in each final model. The parameter values for each of the final models were estimated by the maximum likelihood method. In case of significant differences, odds ratios (OR) and their 95% confidence intervals (95% CI) were calculated from the model’s coefficients and their standard deviations.

A two-stage, two-sided parallel group procedure with an overall type I error of 0.05 was used to test the primary hypothesis of a difference in the probabilities for the two arms in this study, with a sample size of 340 patients (170 patients in each group) needed to achieve 90% power for detecting a difference of 13% in the live birth rate.

## Results

### Study population

**Fig 1:**
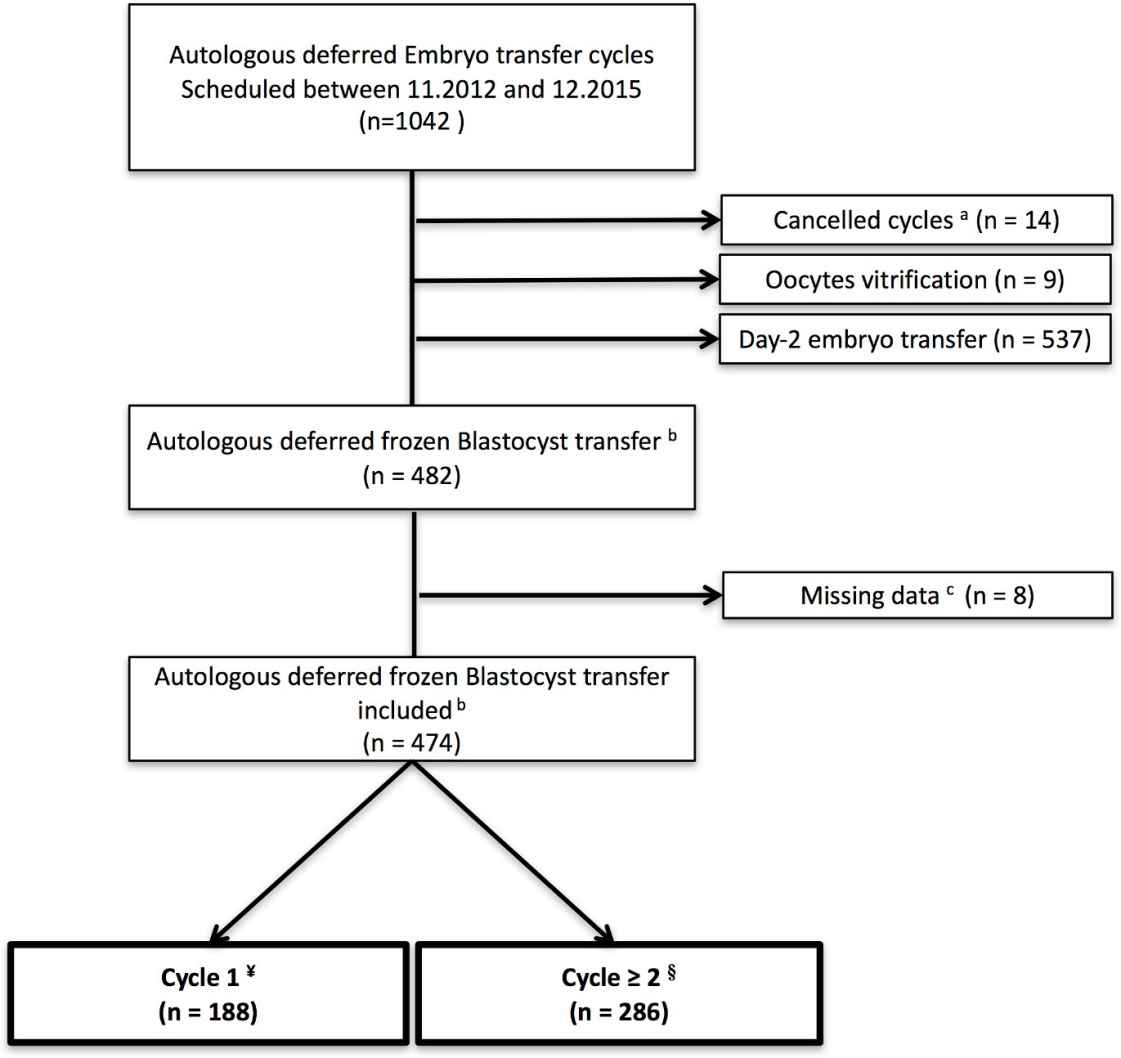
Patient inclusion flowchart. ^a^ Cancelled cycles: poor response; personal or medical (e.g. non-gynecological) reasons. ^b^ Only the first embryos transferred immediately after the COS were retained for the study. ^c^ Missing data: quality of embryo; pregnancy outcomes. ^¥^ Cycle 1: Deferred frozen blastocyst transfer performed within the first menstrual cycle. ^§^ Cycle ≥ 2: Deferred frozen blastocyst transfer performed following one or more menstrual cycles.

The process for our cohort selection is detailed in Fig 1. Overall, 474 autologous deferred FBT were analyzed in this study. There were 188 FBT that were performed within the first menstrual cycle following the COS (‘cycle 1’) and 286 FBT that were performed following one or more menstrual cycles (‘cycle ≥ 2’).

### Patient characteristics

The baseline characteristics are presented in Table 1, revealing comparable characteristics in the two study groups. The mean age was 33.74 ± 4.39 and 33.94 ± 4.39 for the ‘cycle 1’group and the ‘cycle ≥ 2’group, respectively (p = 0.629).

**Table 1:** Baseline characteristics.

	Cycle 1 (n = 188)	Cycle ≥ 2 (n = 286)	*p*-value
**Age (y.o.)**	33.74 ± 4.39	33.94 ± 4.39	0.629 ^t^
**Age > 35 y.o. at retrieval**	65 (34.57)	103 (36.01)	0.752 ^k^
**Smoker**	17 (9.04)	29 (10.14)	0.689 ^k^
**BMI**	23.65 ± 4.35	23.15 ± 4.00	0.237 ^t^
**BMI > 30**	20 (10.64)	20 (6.99)	0.163 ^k^
**Type of infertility**			0.920 ^k^
Primary	131 (68.68)	198 (69.23)	
Secondary	57 (30.32)	88 (30.77)	
**Length of the infertility (y.o.)**	4.32 ± 2.46	4.54 ± 2.27	0.366 ^t^
**Cause of the infertility**			0.461 ^k^
Ovulation disorder	17 (9.04)	30 (10.49)	
Male factor	50 (26.60)	76 (26.57)	
Tubal factor	30 (15.96)	27 (9.44)	
Endometriosis	33 (17.55)	47 (16.44)	
Idiopathic	27 (14.36)	48 (16.78)	
Diminished ovarian reserve	2 (1.06)	5 (1.75)	
More than one cause	29 (15.43)	53 (18.53)	
**Numbers of previous IVF/ICSI attempts**			0.131 ^k^
1	106 (56.38)	171 (59.79)	
2	29 (15.43)	56 (19.58)	
> 3	53 (28.19)	59 (20.63)	
**Ovarian reserve**			
FSH (U/L)	6.29 ± 1.86	6.34 ± 1.68	0.747 ^t^
FSH > 8	22 (11.70)	36 (12.59)	0.777 ^k^
AMH (ng/mL)	5.80 ± 4.19	5.58 ± 4.06	0.576 ^t^
AMH < 2	25 (13.30)	37 (12.94)	0.920 ^k^
AFC	20.56 ± 10.65	21.20 ± 11.69	0.547 ^t^
AFC < 10	23 (12.23)	35 (12.24)	0.999 ^k^
**Indications for FBT**			0.154 ^k^
Risk of OHSS	81 (43.09)	128 (44.76)	
Elevated progesterone or inadequate endometrium	11 (5.85)	30 (10.49)	
Endometriosis	36 (19.15)	55 (19.23)	
Two or more ART failures	47 (25.00)	49 (17.13)	
Autoimmune disease and/or a high risk of thromboembolic disease	13 (6.91)	24 (8.39)	

y.o., years old; BMI, body mass index; IVF/ICSI, in vitro fertilization/ intra-cytoplasmic sperm injection; AFC, antral follicle count; AMH, anti-Müllerian hormone; FSH, follicle stimulating hormone; FBT, frozen blastocyst transfer; OHSS, ovarian hyperstimulation syndrome; ART, assisted reproductive technologies. Data are the mean ± standard error or n (%), unless specified otherwise.

^t^ Student’s t-test

^k^ Pearson’s chi-square test

### ART characteristics

The ART characteristics are presented in Table 2. There were no significant differences between the groups in regard to the COS characteristics and the ovarian response to ovarian stimulation. The endometrial preparation, such as the route of the estradiol administration for the endometrial preparation, the progesterone level before the transfer, and the proportion of individuals with an endometrial thickness equal to or greater than 10 mm were comparable for the two groups. There were no significant differences between the two groups with regard to the number of embryos transferred and the embryo quality.

**Table 2:** ART characteristics and outcomes.

	Cycle 1 (n = 188)	Cycle ≥ 2 (n = 286)	*p*-value
**Stimulation protocol**			0.771^k^
GnRH antagonist	177 (94.16)	265 (92.66)	
Long GnRH agonist	8 (4.27)	14 (4.89)	
Short agonist	3 (1.57)	7 (2.45)	
**Duration of the stimulation (days)**	9.19 ± 1.37	9.41 ± 1.26	0.087 ^t^
**Total dose of injected gonadotropins (IU)**	1,963.91±707.48	2,077.84±784.64	0.102 ^t^
**Type of triggering**			0.033 ^k^
hCG	25 (13.30)	60 (20.98)	
GnRH agonist	163 (86.70)	226 (79.02)	
**Peak estradiol levels (pg/mL)^a^**	2,707.97±1481.29	2,839.61±1631.70	0.365 ^t^
**Peak progesterone levels (ng/mL)^a^**	0.85 ± 0.48	0.95 ± 0.73	0.101 ^t^
**Number of oocytes retrieved**	15.85 ± 7.55	15.38 ± 6.87	0.494 ^t^
**Route of estradiol for endometrial preparation**			0.060 ^k^
Transdermal	175 (93.08)	251 (87.76)	
Oral	13 (6.92)	35 (12.24)	
**Endometrial thickness ≥ 10 mm**	61 (32.45)	70 (24.48)	0.057 ^k^
**Progesterone before transfer (ng/mL)**	0.40 ± 0.25	0.43 ± 0.25	0.230 ^t^
**Number of embryos transferred**			0.574 ^k^
1	185 (98.40)	282 (98.60)	
2	3 (1.60)	4 (1.40)	
**Good quality embryo transfer ^b^**	165/188 (87.77)	258/286 (90.21)	0.399 ^k^
**Clinical pregnancy rate**	82/188 (43.62)	125/286 (43.71)	0.999 ^k^
**Miscarriage rate**	20/82 (24.39)	37/125 (29.60)	0.413 ^k^
**Ongoing pregnancy rate**	62/188 (32.98)	88/286 (30.77)	0.610 ^k^
**Live birth rate**	59/188 (31.38)	85/286 (29.72)	0.696 ^k^
**Gestational age at delivery ^‡^, WGA**	39.56 ± 2.00	39.33 ± 2.47	0.564 ^t^
**Number of twin pregnancies**	0/59 (0.00)	3/85 (3.43)	0.203 ^k^
**Term birth < 37 GA^‡^**	4/59 (6.78)	7/85 (8.24)	0.505 ^k^
**Mean birth weight ^‡^, g**	3,436.55 ± 508.05	3,234.23 ± 606.30	0.045 ^t^
**Birth weight ≥ 4,000 g ^‡^**	0/59	0/82	1.000
**Birth weight < 2,500 g ^‡^**	2/59 (3.39)	6/82 (7.32)	0.271 ^k^
**Z-score ^‡^**	0.14 ± 0.94	-0.21 ± 1.13	0.107 ^t^

IVF/ICSI, in vitro fertilization/ intra-cytoplasmic sperm injection; WGA, weeks of gestational age; ART, assisted reproductive technology

^a^ On the day of triggering

^b^ A good-quality embryo was defined as a B3-B4 or B5 embryo ≥ BB (AA, AB, BA, BB) according to the grading scale proposed by Gardner

^t^ Student’s t-test

^k^ Pearson’s chi-square test

^‡^ Among singletons

Data are the mean ± standard error or n (%), unless specified otherwise

### ART outcomes

We found no significant differences in the live birth rates according to the number of cycles after the COS (59/188 (31.38%) for the ‘cycle 1’ group vs. 85/286 (29.72%) for the ‘cycle ≥ 2’ group, respectively, p = 0.696) (Table 2). There were no significant differences between the ‘cycle 1’ and the ‘cycle 2’ groups in terms of clinical pregnancy rates (82/188 (43.62%) vs. 125/286 (43.71%); p = 0.999), miscarriage rates (20/82 (24.39%) vs. 37/125 (29.60%); p = 0.413), and ongoing pregnancy rates (62/188 (32.98%) vs. 88/286 (30.77%); p = 0.610, respectively).

### Obstetrical, delivery, and neonatal outcomes

The mean birth weight was significantly lower for the ‘cycle ≥ 2’ group as compared to the ‘cycle 1’group (3,436.55 ± 508.05 vs. 3,234.23 ± 606.30; respectively, p = 0.045). However, there was no significant difference in the Z-scores, the number of low birth weights (< 2,500 g), or the number of high birth weights (≥ 4,000 g) (Table 2).

The gestational age at delivery, the number of twin pregnancies, and the number of premature deliveries (term birth < 37 GA) were comparable between the two groups (Table 2).

### Variables independently associated with the live birth rate

A univariate and multivariate analysis was performed to identify variables independently associated with the live birth rate (Table 3). The multivariate model included the women’s age (> 35 y.o. versus ≤ 35 y.o.), the IVF/ICSI rank, the peak estradiol levels (pg/mL) at triggering, the type of triggering, the embryo quality (a good-quality embryo–as defined in the Materials and Methods section- vs. an embryo of lesser quality), and the cycle of transfer (‘cycle ≥ 2’ vs. ‘cycle 1’). After multivariate analysis, the women’s age (> 35 y.o. versus ≤ 35 y.o.) had the most substantial statistically significant impact on the live birth rate (OR: 0.45 [0.29–0.71]; p = 0.001). Performing the FBT during ‘cycle 1’ versus subsequent menstrual cycles (‘cycle ≥ 2’) after oocyte retrieval did not have a significant effect on the live birth rate.

**Table 3:** Factors affecting the live birth rate after frozen-thawed blastocyst transfer: Logistic regression analysis of the risk.

	*Univariate logistic regression analysis*			Multiple logistic regression analysis*		
Parameters	Odds ratio	95% CI	*p*-value	Odds ratio	95% CI	*p*-value
**Age > 35 y.o. at retrieval**	0.47	0.30–0.72	0.001	0.48	0.31–0.75	0.001
**Smoker**	1.22	0.63–2.36	0.560			
**BMI > 30**	0.40	0.34–1.52	0.393			
**Secondary vs. primary infertility**	0.76	0.49–1.17	0.211			
**IVF/ICSI rank^£^**			0.353			0.759
One previous IVF/ICSI cycle vs. no previous IVF/ICSI cycle	0.94	0.56–1.60	0.831	1.02	0.59–1.75	0.942
≥ 2 previous IVF/ICSI cycles vs. no previous IVF/ICSI cycle	0.69	0.42–1.14	0.151	0.83	0.49–1.40	0.486
**Patient’s ovarian reserve:**						
Day-3 FSH > 8 (IU/L)	0.82	0.44–1.53	0.528			
AMH < 2 ng/mL	0.84	0.46–1.53	0.574			
AFC < 10	1.20	0.67–2.15	0.536			
**Peak estradiol levels (pg/mL)** **at triggering**			0.362			0.546
< 1,500^a^	1.28	0.73–2.26	0.394	1.22	0.68–2.11	0.500
> 2,500^a^	1.42	0.88–2.28	0.154	1.31	0.81–2.14	0.272
**Type of triggering:**						
hCG vs. GnRH agonist	1.48	0.86–2.57	0.158	1.38	0.79–2.41	0.258
**Nb. of embryos transferred (2 vs. 1)**	0.89	0.17–4.64	0.890			
**Good quality embryo transfer ^b^**	0.52	0.25–1.08	0.080	1.52	0.95–2.41	0.078
**Cycle of transfer (‘cycle ≥ 2’ vs. ‘1’)**	0.93	0.62–1.39	0.726	0.94	0.62–1.42	0.770
**Estradiol treatment**						
Oral vs. transdermal	0.82	0.42–1.61	0.571			
**Endometrial thickness ^c^**	1.03	0.93–1.14	0.600			
**Progesterone ^c^**	0.68	0.30–1.53	0.350			

y.o., years old; AMH, anti-Müllerian hormone; AFC, antral follicle count; FSH, follicle stimulating hormone; IVF/ICSI, in vitro fertilization / intracytoplasmic sperm injection; Nb, number

* Age (> 35 y.o.), IVF/ICSI rank, Peak estradiol levels (pg/mL) at triggering, Type of triggering, Embryo quality, and FBT cycle (cycle ≥ 2 vs. cycle 1) were included in the multiple logistic regression model.

^£^ - The number of previous IVF/ICSI cycles is defined as the number of previous controlled ovarian stimulations leading to at least one embryo transfer with no pregnancy being obtained.

^a^—Peak estradiol levels (pg/mL) at triggering comprised between 1,500 and 2,500 IU were considered as the reference.

^b^—A good-quality embryo was defined as a B3-B4 or B5 embryo ≥ BB according to the grading scale proposed by Gardner

^c^- on the 1^st^ day of the progesterone administration

## Discussion

### The main finding

The aim of our study was to investigate whether the interval between ovarian stimulation and frozen-thawed blastocyst transfer affects reproductive outcomes in the setting of a Def-ET strategy. By univariate and multivariate analysis, our results found comparable live birth rates whether the frozen-thawed blastocyst transfer was performed during the first menstrual cycle or during subsequent menstrual cycles after the oocyte retrieval, which is reassuring for obstetrical and neonatal issues.

### Strengths and limitations

To the best of our knowledge, our study is the first to have examined the effect of the number of menstrual cycle between COS and the transfer in the specific case of frozen-thawed blastocyst on live birth rates and obstetrical outcomes. Our results shed new light on practical aspects of the Def-ET strategy and they are applicable to daily clinical practice: the embryo transfer in a Def-ET strategy can be scheduled during the first menstrual cycle following the stimulation, without a reduction in the live birth rate. Our study's large sample size (n = 474) is likely to have limited any selection and statistical bias. Lastly, another strength of our study is that epidemiological variables were prospectively collected through face-to-face interviews prior to the ART.

Despite the precautions taken, our study may nonetheless be subject to certain biases. The present study suffers from the limitation of its design involving a retrospective analysis of a prospective cohort. However, we analyzed a homogenous sample of Def-ET with comparable baseline characteristics, and we focused on blastocyst transfers only. In addition, the use of a multivariate model and a large study sample size served to minimize sources of bias. The choice of performing the embryo transfer during the first cycle or during subsequent cycles after the stimulation was not related to the women’s or the cycle’s characteristics. Rather, it was only contingent on the physical possibility of scheduling the FBT cycle, based on the patient's availability or the center’s workload.

### Interpretation

A number of previous studies have tried to discern whether optimal conditions for embryo transfer are present as of the first cycle after ovarian stimulation. In unadjusted and adjusted analyses of the effect of the timing of frozen embryo transfer on the clinical pregnancy rate, Santos-Ribeiro *et al*. found a borderline significant effect in favor of immediate frozen embryo transfer (unadjusted OR: 0.63, 95% CI [0.41–0.99]; adjusted OR: 0.62, 95% CI: [0.38–1.00]). A mix of cleavage stages and blastocyst stages were included in the analysis [17]. In the same way, focusing on day-3 and day-4 embryo transfers, Lattes *et al*. found -after univariate analysis- that the LBR was significantly higher with frozen embryo transfers performed during ‘cycle 1’ vs. ‘cycle ≥ 2’. However, after adjusting for confounding factors, the authors found that there were no longer any significant differences between embryo transfers performed during the first versus the subsequent menstrual cycles after COS [18]. Interpreting these results is complex due to the high level of heterogeneity in the study populations and particularly in light of the various stages of embryo development that they analyzed. As in our study, Ozgur *et al*. analyzed frozen blastocyst transfer. The LBR after only one menstruation was similar to the LBR for embryo transfers performed after a delay of one or more cycles [19]. In a ‘freeze all’ approach, our study did not find any differences in ART outcomes based on whether the frozen-thawed blastocyst transfer was performed within the first menstrual cycle following the COS or following one or more menstrual cycles. Thus, one can hypothesize that endometrial receptivity is restored after the first withdrawal bleeding. Our findings support those of previously published studies [17–19] regarding pregnancy outcomes.

One fear for couples is that the Def-ET strategy could increase the time to become pregnant as compared to a fresh embryo transfer [28,29]. Our findings confirm that embryo transfers can be performed as of the first menstrual cycle after ovarian stimulation, and that it is not necessary to wait longer after the oocyte retrieval to schedule a frozen embryo transfer. In clinical practice, our findings are of substantial relevance for minimizing the time to become pregnant in the Def-ET strategy and for improving the level of patients’ satisfaction with the ART process.

In regard to obstetrical and neonatal outcomes, in our study we found that the mean birth weight was significantly lower for the ‘cycle ≥ 2’ group as compared to the ‘cycle 1’group. However, there were no significant differences in the Z-scores, which are determined after adjustment for the gestational age and the child’s gender. Moreover, we did not find any significant differences in terms of low birth weights or high birth weights number between the studied groups. All-in-all, these data appear to indicate that there is no effect of the interval between COS and FBT on birth weights.

## Conclusion

Based on our findings, the chances of a live birth after a frozen-thawed blastocyst transfer, as well as the obstetrical and neonatal outcomes, are the same whether the deferred embryo transfer is performed within the first menstrual cycle after oocyte retrieval or during subsequent cycles. Delaying FBT by more than one menstrual cycle can therefore be avoided with a ‘freeze all’ strategy as it does not provide patients with a greater chance of achieving a live birth.
